# OLDER PRIMARY CARE PATIENTS’ ENGAGEMENT WITH STEADI FALL PREVENTION RECOMMENDATIONS

**DOI:** 10.1093/geroni/igac059.3096

**Published:** 2022-12-20

**Authors:** Hiroko Kiyoshi-Teo, Bryanna De Lima, Debbie Cohen, Kerri Winters-Stone, Elizabeth Eckstrom

**Affiliations:** Oregon Health & Science University, Portland, Oregon, United States; Oregon Health & Science University, Portland, Oregon, United States; Oregon Health & Science University, Portland, Oregon, United States; Oregon Health & Science University, Porltand, Oregon, United States; Oregon Health & Science University, Portland, Oregon, United States

## Abstract

The Stopping Elderly Accidents, Deaths, and Injuries (STEADI) initiative is a CDC-disseminated strategy to provide fall prevention recommendations to high risk patients. However, whether older patients engage with fall prevention recommendations is less clear. The aims of this study were to: 1) describe STEADI fall prevention recommendations provided to eligible older patients; and 2) understand patients’ attitudes, confidence and adherence to recommendations. Fall prevention recommendations were identified by chart review (age ≥65 years old; STEADI score ≥4 (high risk); free of severe cognitive impairment). For those who consented to the study, questionnaires measured recollection (Yes/No), feelings (Positive/Negative), overall confidence to reduce fall risks through the recommendations (0–10 scale; 10 = most confident), and adherence to the specific recommendations (Yes/No). Results showed that 30% (n = 458) of eligible older adults received fall prevention recommendations. Among study participants (n = 182; 69% female, age 80.2 ± 7.7 years, STEADI score 7.0 ± 2.6), 73% reported positive reactions to recommendations. Overall confidence to reduce gait/balance-related fall risks through recommendations was 7.52 (n = 137) with adherence to physical therapy at 70% and tai chi at 38%. Overall confidence to reduce blood pressure-related fall risks through recommendations was 7.45 (n = 55) with adherence to adjusting medications at 89% compared to 69% for compression socks. Participants reported varying levels of adherence despite positive reactions and moderate confidence to reduce their fall risks. Additional research is needed to identify barriers and facilitators to improve adherence.

